# THE ASSOCIATION OF CAREGIVER STRAIN AND NEEDS OF CAREGIVERS AND PEOPLE LIVING WITH DEMENTIA

**DOI:** 10.1093/geroni/igad104.3627

**Published:** 2023-12-21

**Authors:** Allison Griswold, Miranda Moore, Joanna Jungermann, Molly Perkins, Alexis Bender

**Affiliations:** Emory University, Atlanta, Georgia, United States; Emory University, Atlanta, Georgia, United States; Emory University, Atlanta, Georgia, United States; Emory University, Atlanta, Georgia, United States; Emory University, Atlanta, Georgia, United States

## Abstract

Caregivers play a vital part of dementia care management and are key to keeping people living with dementia at home. Providing this care can result in caregiver strain, especially among those who live in low or under resourced communities, such as rural or impoverished areas. One instrument for measuring caregiver strain among this population is the BRI caregiver strain instrument, which includes four dimensions of strain including caregiver mastery, relationship strain, health strain and social isolation. To date, little research assessing the validity of the instrument. We assessed the validity using confirmatory factor analysis in a cohort of 930 caregivers providing care to people undergoing assessment for dementia representing four diverse regions in Georgia (Atlanta, Albany, Augusta, and Macon). Then, we examined the associations of the lack of caregiver knowledge of dementia, care recipient function, and challenging behaviors using univariate and multivariate linear regression. We found that the four-factor solution showed good model fit and was appropriate for subsequent analysis with this population. In our regression models, we found that caregiver lack of understanding of dementia significantly predicted caregiver strain (β=2.09, P<.001). When controlling for care recipient function, lack of knowledge decreased in strength, but remained significant (β=1.86, P<.001). When adding challenging behaviors, lack of dementia knowledge lost significance and challenging behaviors became the strongest predictor of caregiver strain (β=2.03, P<.001). Caregiver strain is a multifactorial process and tools to reduce challenging behaviors and increase caregiver knowledge are promising points of intervention early in the diagnostic process.

